# Epidemiology of adenosarcoma and the inverse probability of treatment weighting (IPTW) adjusted survival analysis of lymph node dissection in uterine adenosarcoma

**DOI:** 10.1097/MD.0000000000030607

**Published:** 2022-09-23

**Authors:** Hanjie Hu, Zhewen Wei, Hong Zhao, Guangwen Yuan

**Affiliations:** a Key Laboratory of Gene Editing Screening and R&D of Digestive System Tumor Drugs, Chinese Academy of Medical Sciences and Peking Union Medical College, Beijing, China; b Department of Hepatobiliary Surgery, National Cancer Center/National Clinical Research Center for Cancer/Cancer Hospital, Chinese Academy of Medical Sciences and Peking Union Medical College, Beijing, China; c Department of Gynecological Oncology, National Cancer Center/National Clinical Research Center for Cancer/Cancer Hospital, Chinese Academy of Medical Sciences and Peking Union Medical College, Beijing, China.

**Keywords:** adenosarcoma, inverse probability of treatment weighting, lymph nodes dissection

## Abstract

The objective for the study was to analysis the epidemiology of adenosarcoma, and independent prognostic factors and impact of lymph node dissection (LND) of uterine adenosarcoma. Cases of patients with primary adenosarcoma were obtained from the Surveillance, Epidemiology, and End Results (SEER) database from 2000 to 2016. Overall survival was analyzed by the Kaplan–Meier method and log-rank test. The differences in baseline covariates between the 2 groups were adjusted by inverse probability of treatment weighting method. The prognostic factors were identified by univariate and multivariate Cox regression analysis and hazard ratio and 95% confidence interval (CI) of covariates were also estimated. 1129 patients with pathological primary adenosarcoma between 2000 and 2016 were identified from the surveillance, epidemiology, and end results database. The only 4 patients were male. 1027 patients with primary uterine adenosarcoma, and 53.1% underwent LND and only 3.5% patients were with positive lymph node. Age, marital status, largest tumor size, tumor grade, T stage and chemotherapy were significantly correlated with survival. Race, tumor number, LND, and radiotherapy did not affect overall survival in patients. Inverse probability of treatment weighting-adjusted K-M curve showed that LND did not improve survival and lymph node metastasis (LNM) did not affect survival. The majority of primary adenosarcoma patients are female with high incidence of uterus and rare incidence of distant metastasis. Age, marital status, tumor size, T stage, grade, and chemotherapy are independent prognostic factors of uterine adenosarcoma. LNM was not a significant prognostic risk factor, and LND did not benefit survival.

## 1. Introduction

Adenosarcoma is a rare tumor with a mixture of epithelial and stromal components.^[1–3]^ It occurs almost entirely in the female reproductive system and typically arises from the corpus uterus, rarely from the cervix or ovary.^[2,4,5]^ Adenosarcoma occasionally occurs in extra-uterine and extra-ovarian sites such as the peritoneum, retroperitoneum, bladder, liver or colon, and it is generally assumed that the biological behaviors of extra-uterine adenosarcoma is associated with endometriosis.^[3,6]^ Uterine adenosarcoma accounts for 2% to 5% of all uterine sarcomas.^[7]^

It is well known that sarcoma is a malignant tumor originating from mesenchymal tissue, and adenocarcinoma originated from glandular epithelium. However, adenosarcoma often occur in the glandular epithelium with a mixture of sarcoma components, especially in the uterine muellerian epithelium.^[2,8,9]^ Some literatures still put adenosarcoma in the same category as sarcoma.^[10–12]^ Most uterine adenosarcoma has been described in the literature are of low grade malignancy,^[13,14]^ stage I without sarcomatous overgrowth have a rather good prognosis, with a 5-year overall survival (OS) up to 80%.^[14]^ When the component of the sarcoma is more than a quarter of the volume which called sarcoma overgrowth, it will develop aggressive behavior and is characterized by recurrence and metastasis.^[15–18]^ The latest research reported that relapse was related to histology and leiomyosarcoma had the worst prognosis with the OS of 57.1% and the relapse rate of 71%, followed by low-grade endometrial stromal sarcoma with the relapse rate of 54%.^[19]^ Surgery is the mainstay of treatment for uterine adenosarcoma and chemotherapy and radiotherapy use are not clear.^[20,21]^ The surgery procedure and the effect of lymph nodes dissection (LND) remain controversial.^[20,21]^

In this study, we aim to characterize the epidemiology of adenosarcoma. Then, we aim to identify the independent prognostic factors of uterine adenosarcoma and assess the impact of LND on survival, hoping to make some suggestions for treatment.

## 2. Materials and Methods

### 2.1. Patient cohort

The clinical data of patients with pathological primary adenosarcoma between January 1, 2000 and December 31, 2016 were downloaded from the Surveillance, Epidemiology, and End Results (SEER) database using SEER*Stat software (version 8.3.6). These data were located within the SEER dataset by using histology codes 8933 (International Classification of Disease for Oncology, 3rd edition, ICD-O-3). Only patients who underwent surgery were included. The exclusion criteria included the following: patients younger than 18; patients whose postoperative survival was less than 1 month; and patients whose radiotherapy or chemotherapy was unknown.

### 2.2. Clinical and demographic variables

Data collected from eligible patients included the following: age at diagnosis (median and range), sex (female and male), year of diagnosis (2000–2005, 2006–2011, and 2012–2016), marital status (married, unmarried and unknown), race (white, black, other and unknown), primary tumor site, total number (multiple and single), largest tumor size, tumor grade (grades I–IV and unknown), T stage (T 1–4 and x), metastasis, LND, lymph node status (positive, negative, and unknown), radiotherapy, chemotherapy, and the length of OS time. Frequency analyses and descriptive statistics were performed on the collected data.

### 2.3. Statistical analysis

Continuous data are presented as median and interquartile range (IQR), and categorical data are presented as frequency and percentage. Chi-square tests were used to compare the statistical significance of samples between different variables. OS was analyzed using the Kaplan–Meier method and log-rank tests were used for comparisons between LND group and non-LND (nLND) group.

The observed differences in baseline covariates between the 2 groups were adjusted by using inverse probability of treatment weighting (IPTW) method to reduce the selection bias. The IPTW approach is attempting to mimic a situation in which treatment is randomly allocated to individuals. Factors associated either with the receipt of LND or with OS were included in constructing the models, which included age, marital status, race, tumor number, largest tumor size, grade, pathologic T stage, lymph node status, radiotherapy, chemotherapy. The adjusted Kaplan–Meier curves and log-rank test based on inverse probability weights were computed to compare OS between LND group and nLND group.

Univariate Cox proportional hazards regression analysis was performed for various risk factors using the above methods. Only variables shown to be statistically different through univariate Cox regression analysis were included in multivariate Cox regression analysis to identify the independent prognostic factors and estimate the hazard ratio (HR) and 95% confidence interval (CI) of covariates. The effect of independent factors on survival was further analyzed.

A *P* value of < 0.05 was considered to indicate statistical significance. The best cutoff value was found using the X-tile software (Yale School of Medicine/Pathology/Rimm Lab, New Haven, CT) with the minimum *P* value method. Statistical analyses were performed using IBM SPSS Statistics 22.0 (IBM, Armonk, NY) for R (version 3.6.2) and RStudio (RStudio, Vienna, Austria).

## 3. Results

### 3.1. Patient characteristics

**Figure 1.**
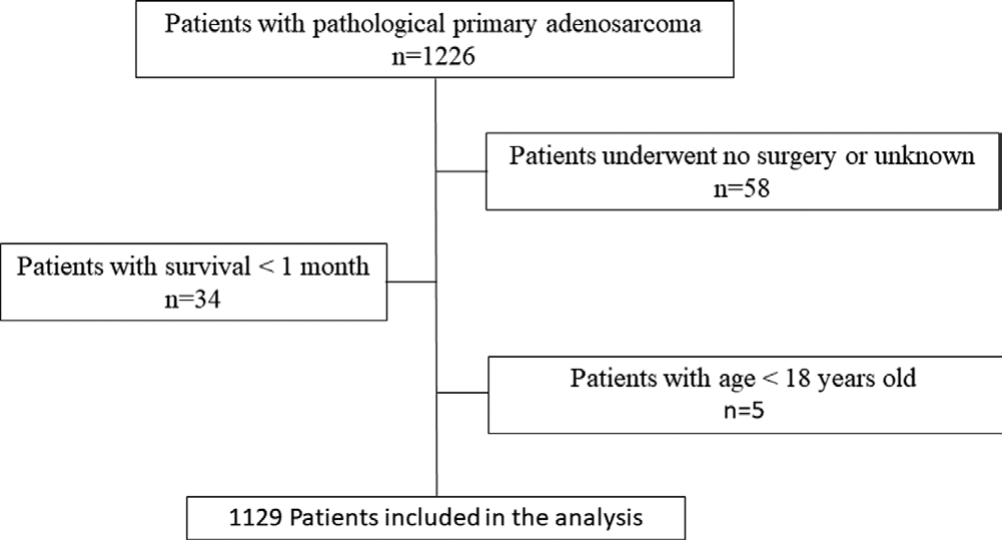
The flowchart of screening of patients in SEER database.

This study was conducted in accordance with the Declaration of Helsinki (as revised in 2013). All data were retrieved from the public SEER database, so this study was deemed exempt from Ethics Committee of National Cancer Center/Cancer Hospital, Chinese Academy of Medical Sciences and Peking Union Medical College. A total of 1129 patients with pathological primary adenosarcoma between 2000 and 2016 were identified from the SEER database (Fig. 1). Of these patients who received surgery, the median follow-up time in the surgery was 57 months with an IQR of 20 to 113 months. Their median age was 56 years old with an IQR of 46 to 67. The vast majority (99.6%) were female, and only 4 patients were male. Female reproductive organs (96.0%) were the most common primary sites and uterus (91.0%) accounted for the majority, and other primary sites were <5%. The distant metastasis was <1% from 2010. The baseline characteristics of all eligible patients are listed in Table 1. The study cohort had a good survival that 5-year survival was 71.9%, median OS was not reached.

**Table 1:** Characteristics of pathological primary adenosarcoma patients who underwent operation.

Variables	Overall, n = 1129 (%)
Age (yr)	
Median	56
Range (IQR)	46–67
<56	548 (48.5%)
≥56	581 (51.5%)
Sex	
Female	1125 (99.6%)
Male	4 (0.4%)
Year of diagnosis	
2000–2005	367 (32.5%)
2006–2011	389 (34.5%)
2012–2016	373 (33.0%)
Marital status	
Yes	563 (49.9%)
No	518 (45.9%)
Unknown	48 (4.3%)
Race	
White	865 (76.7%)
Black	139 (12.3%)
Other*	120 (10.6%)
Unknown	5 (0.4%)
Primary site	
Uterus	1027 (91.0%)
Ovary	43 (3.8%)
Other female reproductive organs	13 (1.2%)
Peritoneum and retroperitoneum	15 (1.3%)
Soft tissue	17 (1.4%)
Gastrointestinal tract	8 (0.7%)
Breast	1 (0.1%)
Lung	3 (0.3%)
Kidney	1 (0.1%)
Extrahepatic bile duct	1 (0.1%)
Total number	
Mean	1.28
Range	1–7
Multiple lesions	
Yes	244 (21.6%)
No	885 (78.4%)
Largest tumor size (mm)	
Present	737 (65.3%)
Missing	392 (34.7%)
Mean	68.33
Range	1–420
Grade	
Grade I	142 (12.6%)
Grade II	206 (18.2%)
Grade III	72 (6.4%)
Grade IV	147 (13.0%)
Unknown	562 (49.8%)
T stage	
T1	319 (28.3%)
T2	35 (3.1%)
T3	17 (1.5%)
T4	2 (0.2%)
Tx	756 (67.0%)
Metastasis (2010+)†	
Bone	0
Brain	0
Liver	1 (0.1%)
Lung	2 (0.2%)
Lymph node dissection	
Yes	586 (51.9%)
No	543 (48.1%)
Lymph node status	
Positive	22 (3.8%)
Negative	544 (92.8%)
Unknown	20 (3.4%)
Radiotherapy	
Yes	197 (17.4%)
No	932 (82.6%)
Radiotherapy sequence	
Prior surgery	7 (0.6%)
Prior and after surgery	3 (0.3%)
After surgery	187 (16.6%)
Chemotherapy	
Yes	162 (14.3%)
No	967 (85.7%)
Follow-up time (mo)	
Median	57
Range (IQR)	20–113

IQR: interquartile range.

* Others: American Indian/AK Native, Asian/Pacific Islander.

† Metastasis (2010+): the SEER database began incorporating metastasis data from 2010.

### 3.2. Uterine adenosarcoma patient characteristics

One thousand twenty-seven patients with primary uterus adenosarcoma, and the median age was 56 with an IQR of 46 to 67. Near half (49.8%) patients were married. 78.0% patients had single tumor lesion and the mean largest tumor size was 63.96 mm with a range of 1 to 300. Over half patients (53.1%) underwent lymph node dissection and only 3.5% patients were with positive lymph node. Patient characteristics are listed in Table 2.

**Table 2:** Clinical features between uterus adenosarcoma patients in lymph node dissection and non-dissection group.

Variables	All n = 1027(%)	Lymph node dissection		
Yes (n = 545[53.1%])	No (n = 482[46.9%])	*P* value
Age (yr)				0.610
Median	56	57	55.5	
Range (IQR)	46–67	47–66	46–67	
Age at diagnosis (yr)				0.277
<56	495 (48.2%)	254 (46.6%)	241 (50.0%)	
≥56	532 (51.8%)	291 (53.4%)	241 (50.0%)	
Marital status				0.037
Yes	511 (49.8%)	291 (53.4%)	220 (45.6%)	
No	469 (45.7%)	233 (42.8%)	236 (49.0%)	
Unknown	47 (4.6%)	21 (3.9%)	26 (5.4%)	
Race				0.619
White	783 (76.2%)	417 (76.5%)	366 (75.9%)	
Black	126 (12.3%)	61 (11.2%)	65 (13.5%)	
Other*	113 (11.0%)	64 (11.7%)	49 (10.2%)	
Unknown	5 (0.5%)	3 (0.6%)	2 (0.4%)	
Multiple lesions				0.771
Yes	226 (22.0%)	118 (21.7%)	108 (22.4%)	
No	801 (78.0%)	427 (78.3%)	374 (77.6%)	
Tumor size				0.030
Present	666 (64.8%)	370 (67.9%)	296 (61.4%)	
Missing†	361 (35.2%)	175 (32.1%)	186 (38.6%)	
Largest tumor size (mm)				0.685
≤48	271 (40.7%)	148 (40%)	123 (41.6%)	
>48	395 (59.3%)	222 (60%)	173 (58.4%)	
Grade				0.059
Grade I	127 (12.4%)	56 (10.3%)	71 (14.7%)	
Grade II	190 (18.5%)	99 (18.2%)	91 (18.9%)	
Grade III	58 (5.6%)	35 (6.4%)	23 (4.8%)	
Grade IV	139 (13.5%)	85 (15.6%)	54 (11.2%)	
Unknown	513 (50.0%)	270 (49.5%)	243 (50.4%)	
T stage				0.927
T1	314 (30.6%)	164 (30.1%)	150 (31.1%)	
T2	19 (1.9%)	10 (1.8%)	9 (1.9%)	
T3	10 (1.0%)	4 (0.7%)	6 (1.2%)	
T4	2 (0.2%)	1 (0.2%)	1 (0.2%)	
Tx	682 (66.4%)	366 (67.2%)	316 (65.6%)	
Lymph node status				-
Positive	19 (3.5%)	19 (3.5%)	0	
Negative	509 (93.4%)	509 (93.4%)	0	
Unknown	17 (3.1%)	17 (3.1%)	0	
Radiotherapy				<0.001
Yes	176 (17.1%)	125 (22.9%)	51 (10.6%)	
No	851 (82.9%)	420 (77.1%)	431 (89.4%)	
Radiotherapy sequence				<0.001
Prior surgery	6 (3.4%)	4 (3.2%)	2 (3.9%)	
Prior and after surgery	3 (1.7%)	2 (1.6%)	1 (2.0%)	
After surgery	167 (94.9%)	119 (95.2%)	48 (94.1%)	
Chemotherapy				0.007
Yes	126 (12.3%)	81 (14.9%)	45 (9.3%)	
No	901 (87.7%)	464 (85.1%)	437 (90.7%)	
Follow-up time (mo)				0.014
Median	58	64	49.5	
Range (IQR)	20–113	22.5–118	18–106.25	

IQR = interquartile range.

* Others: American Indian/AK Native, Asian/Pacific Islander.

† Missing: excluded in the survival analysis.

### 3.3. Prognostic factors of uterine adenosarcoma patients

Univariate and multivariate analyses were conducted to identify the independent prognostic factors of patients with uterine adenosarcoma. Univariate and multivariate Cox regression analysis demonstrated that age, marital status, largest tumor size, tumor grade, T stage and chemotherapy were significantly correlated with survival. An age of 56 or greater (HR 1.897; 95% CI, 1.394–2.581; *P* < .001), unmarried (HR 1.375; 95% CI, 1.036–1.825; *P* = .028), largest tumor size > 48 mm (HR 1.685; 95% CI, 1.226–2.316; *P* = .001), grade III (HR 2.533; 95% CI, 1.301–4.934; *P* = .006) and IV (HR 2.585; 95% CI, 1.419–4.709; *P* = .002), T2 (HR 3.388; 95% CI, 1.641–6.994; *P* = .001) and T3 (HR 6.957; 95% CI, 3.254–14.876; *P* < .001), and chemotherapy (HR 1.827; 95% CI, 1.291–2.586; *P* = .001) were significantly correlated with worse OS. Race, tumor number, LND, and radiotherapy did not affect OS in patients (Table 3).

**Table 3:** Independent prognostic factors of uterus adenosarcoma patients.

	Univariate analysis		Multivariate analysis	
	HR (95% CI)	*P* value	HR (95% CI)	*P* value
Age (yrs)				
<56	1		1	
≥56	2.026 (1.509–2.719)	<.001	1.897 (1.394–2.581)	<.001
Marital status				
Yes	1		1	
No	1.496 (1.134–1.974)	.004	1.375 (1.036–1.825)	.028
Unknown	1.730 (0.898–3.332)	.101	1.598 (0.821–3.112)	.168
Race				
White	1			
Black	1.192 (0.822–1.728)	.354		
Other*	0.912 (0.602–1.382)	.665		
Unknown	0.000 (0.000–1.267)	.946		
Multiple lesions				
No	1			
Yes	1.242 (0.916–1.684)	.164		
Largest tumor size (mm)				
≤48	1		1	
>48	2.482 (1.834–3.359)	<.001	1.685 (1.226–2.316)	.001
Grade				
Grade I	1		1	
Grade II	1.262 (0.672–2.373)	.469	1.264 (0.672–2.382)	.469
Grade III	5.801 (3.100–10.853)	<.001	2.533 (1.301–4.934)	.006
Grade IV	3.543 (1.976–6.356)	<.001	2.585 (1.419–4.709)	.002
Unknown	1.278 (0.724–2.257)	.397	1.141 (0.644–2.019)	.651
T stage				
T1	1		1	
T2	3.466 (1.703–7.051)	.001	3.388 (1.641–6.994)	.001
T3	11.911 (5.815–24.399)	<.001	6.957 (3.254–14.876)	<.001
T4	4.065 (0.560–29.495)	.165	2.523 (0.336–18.931)	.368
Tx	1.155 (0.826–1.615)	.398	1.370 (0.975–1.925)	.070
Lymph node dissection				
Yes	1			
No	0.943 (0.718–1.238)	.673		
Radiotherapy				
No	1		1	
Yes	1.746 (1.300–2.346)	<.001	1.036 (0.753–1.426)	.826
Chemotherapy				
No	1		1	
Yes	2.382 (1.732–3.276)	<.001	1.827 (1.291–2.586)	.001

CI = confidence interval, HR = hazard ratio.

* Others: American Indian/AK Native, Asian/Pacific Islander.

### 3.4. The effect of LND on survival

**Figure 2.**
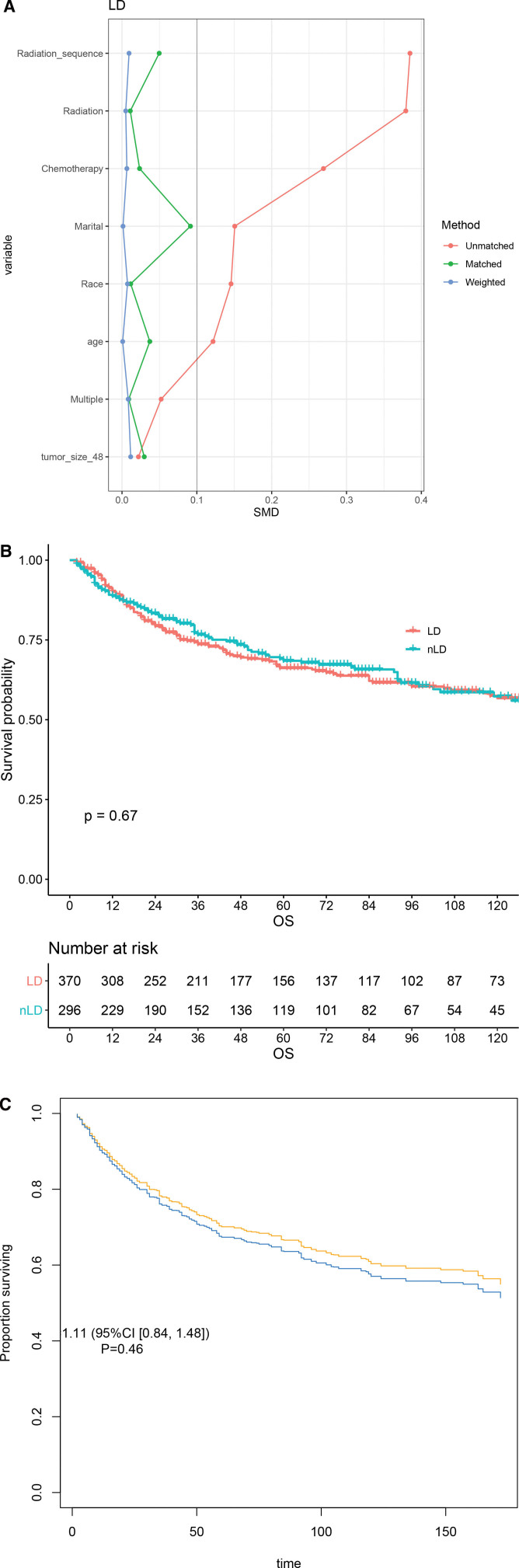
IPTW-adjusted study in LND and nLND groups. The adjustment (A) and the survival before (B) and after (C) IPTW. IPTW = inverse probability of treatment weighting, LND = lymph nodes dissection, nLND = non-LND.

To clarify this issue, we matched factors associated to OS to eliminate differences between LND and nLND groups. Age, marital, race, multi-disease, tumor size, radiation, radiation sequence, and chemotherapy were included. The cut-off value of age was the median age and the cut-off value of largest tumor size was 48 mm which was calculated by X-tile software. In order to make the results more accurate, the cases with the above variables missing were removed in this section. A total of 666 patients were eventually enrolled in this part of the study. Among them, 370 (55.6%) underwent LND. By observing standardized mean difference changes, we can confirm that IPTW effectively balances the baseline (Fig. 2A). Before adjustment was done, OS of both LND and nLND groups were not reached, and there was no statistical significance (*P* = .67, Fig. 2B). IPTW-adjusted K-M curve showed that LND did not improve survival even after balance (Fig. 2C).

**Figure 3.**
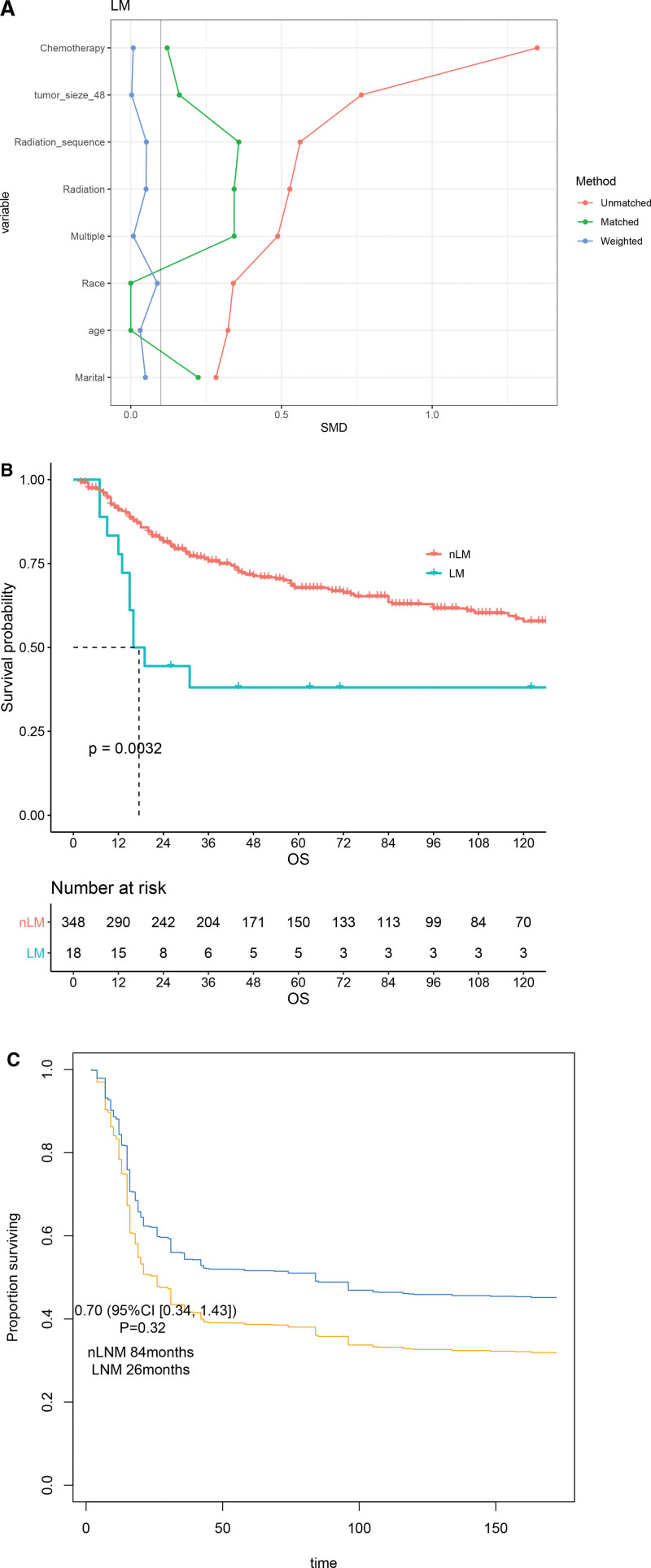
IPTW-adjusted study in LNM and nLNM groups. The adjustment (A) and the survival before (B) and after (C) IPTW. IPTW = inverse probability of treatment weighting, LNM = lymph node metastasis, nLNM = non-LNM.

We further attempted to analyze the impact of lymph node metastasis (LNM) on survival in UAS. Similarly, we balance the above factors (Fig. 3A). Among 370 LND patients, only 18 (4.9%) were pathology confirmed LNM. Before IPTW, median OS of LNM was significant worse than nLNM group (17.5 m vs. NA, *P* = .003, Fig. 3B). However, survival between groups had not statistically significance after adjustment (Fig. 3C).

## 4. Discussion

Adenosarcoma is a rare tumor, which lacks large sample research and treatment experience. It has a good prognosis, but a significant proportion cases still show aggressive behavior.^[17,18]^ The data in SEER show that it often occurs in middle age (IQR 46–67), the overwhelming majority are women (99.6%). 91.0% of the adenosarcoma occurs in uterus, then it is in other parts of female reproductive systems including ovary and others positive lymph node and distant metastases are rare.

Adenosarcoma in female reproductive system is also called mullerian adenosarcoma, which was first reported in 1974.^[8]^ At present, there are no specific guidelines of this disease. International Federation of Gynecology and Obstetrics uses the staging system of endometrial stromal sarcoma for uterine adenosarcoma.^[22]^ Surgical resection is also the most effective treatment for uterine adenosarcoma.^[4]^ LND is a traditional operation of tumor surgery, and routine LND is generally not recommended for sarcomas. But in our study, over a half (55.6%) patients with uterine adenosarcoma underwent LND. Unfortunately, the result indicates that no matter the adjustment was taken, the survival of LND group was not better than the other. We also found this type of tumor to be less prone to LNM (4.9%). LNM showed a poorer prognosis at first, but no statistical significance remained after IPTW. A similar study was published in 2017 by Machida,^[23]^ the study included 877 patients from SEER database who were diagnosed with uterine adenosarcoma and underwent LND, among them 29 had LNM. Similarly, He also found that LNM was a prognostic risk factor. However, multivariate COX method was not effective in removing confounders. Machida also gave a systematic review, LNM was present in 4% of its 230 included cases, and 131 (56%) underwent LND. The rates of LND and LNM were consistent with our study. We used IPTW to balance the baseline and found that LNM may not be a significant prognostic risk factor, which will require more research in the future. Brandon-Luke^[21]^ reported a study based on National Cancer Data Base in 2016. Only 36/1176 (3.1%) had LNM, and was not significantly associated with survival, which met our findings. Anyway, judging by the evidence so far, uterine adenosarcoma has a low tendency of LNM, and LNM has unclear effect on prognosis. While the surgeons chose LND in more than half of the surgeries, which certainly increases the risk of surgical trauma without definite benefit. In view of the above we do not recommend routine LND during uterine adenosarcoma surgery.

Due to the rarity of this disease, its risk factors are not clearly reported. In our cohort, age, marital status, tumor size, T stage, grade and chemotherapy were independent risk factors of uterine adenosarcoma. Sarcomatous overgrowth predicts a poor prognosis of uterine adenosarcoma, which was generally accepted.^[24,25]^ Nathenson^[25]^ studied from 165 patients’ cohort and found that the recurrence rate of UAS with sarcoma overgrowth can be as high as 69.8%, but the rate of other group recurred was only 20.8%. Age may be another risk factor,^[10,18,25]^ but this conclusion may lack specificity. We did not detect statistical significance in multivariate Cox regression. Size was not associated with poor prognosis,^[22,25]^ but myometrial invasion can reduced survival.^[14,25]^ Uterine adenosarcoma generally had better survival than adenosarcoma occurring in the ovary or pelvic cavity.^[25]^ Adenosarcoma of other parts and male were very rare, lack of effective treatment experience. A man with primary liver adenosarcoma was reported in 2020, died due to recurrence after only 31 months after surgery.^[12]^ The adjuvant chemotherapy was usually referring to uterine sarcomas, but the effect of neither chemotherapy nor radiotherapy was definite.^[20,21]^ Despite the different origins, available evidence suggested that the International Federation of Gynecology and Obstetrics staging plays a role in prognosis in uterine adenosarcoma,^[25]^ which was consistent with our conclusion.

This study has some limitations. Firstly, there are many missing variables in the SEER database which we cannot include in analysis. We did not conduct an in-depth study of extra-uterine adenosarcoma due to its low incidence. Also, the sample size difference between groups was too large. Despite this, we still found some problems with the previous surgical methods of this disease and made some suggestions. The incidence of the disease is low, and further studies based on a larger real-word sample are needed.

## 5. Conclusion

The majority of primary adenosarcoma patients are female with high incidence of uterus and rare incidence of distant metastasis. Age, marital status, tumor size, T stage, grade and chemotherapy are independent prognostic factors of uterine adenosarcoma. Further, LNM is not a significant prognostic risk factor of uterine adenosarcoma, and LND did not benefit survival.

## Author contributions

HH and ZW analyzed the data and were major contributors in writing the manuscript. HZ and GY provided technical guidance for the research. All authors read and approved the final manuscript.

**Conceptualization:** Guangwen Yuan, Hanjie Hu, Zhewen Wei.

**Data curation:** Hanjie Hu, Zhewen Wei.

**Formal analysis:** Hanjie Hu, Zhewen Wei.

**Methodology:** Hanjie Hu, Zhewen Wei.

**Project administration:** Guangwen Yuan, Hong Zhao.

**Software:** Hanjie Hu, Zhewen Wei.

**Writing – original draft:** Hanjie Hu, Zhewen Wei.

**Writing – review & editing:** Guangwen Yuan, Hanjie Hu, Hong Zhao, Zhewen Wei.

## References

[1] Clement PB Scully RE. Mullerian adenosarcoma of the uterus: a clinicopathologic analysis of 100 cases with a review of the literature. Hum Pathol. 1990;21:363–81. 2156771 10.1016/0046-8177(90)90198-e

[2] D’Angelo E Spagnoli LG Prat J. Comparative clinicopathologic and immunohistochemical analysis of uterine sarcomas diagnosed using the World Health Organization classification system. Hum Pathol. 2009;40:1571–85. 19540555 10.1016/j.humpath.2009.03.018

[3] Meguro S Yamazaki S Matsushima S. A case of a primary hepatic so-called adenosarcoma with heterotopic ossification: possibly of biliary adenofibroma origin. Hum Pathol. 2018;73:108–13. 29079182 10.1016/j.humpath.2017.10.009

[4] Nathenson MJ Ravi V Fleming N Wang WL Conley A. Uterine Adenosarcoma: a Review. Curr Oncol Rep. 2016;18:68. 27718181 10.1007/s11912-016-0552-7

[5] Ulrich UA Denschlag D. Uterine adenosarcoma. Oncol Res Treat. 2018;41:693–6. 30326467 10.1159/000494067

[6] Mandato VD Torricelli F Mastrofilippo V Valli R Aguzzoli L La Sala GB. Primary extra-uterine and extra-ovarian mullerian adenosarcoma: case report and literature review. BMC Cancer. 2018;18:134. 29402239 10.1186/s12885-018-4037-y PMC5800024

[7] D’Angelo E Prat J. Uterine sarcomas: a review. Gynecol Oncol. 2010;116:131–9. 19853898 10.1016/j.ygyno.2009.09.023

[8] Clement PB Scully RE. Müllerian adenosarcoma of the uterus. A clinicopathologic analysis of ten cases of a distinctive type of Mullerian mixed tumor. Cancer. 1974;34:1138–49. 4371193 10.1002/1097-0142(197410)34:4<1138::aid-cncr2820340425>3.0.co;2-9

[9] Huang PS Chang WC Huang SC. Müllerian adenosarcoma: a review of cases and literature. Eur J Gynaecol Oncol. 2014;35:617–20. 25556263

[10] Nathenson MJ Conley AP. Prognostic factors for uterine adenosarcoma: a review. Expert Rev Anticancer Ther. 2018;18:1093–100. 30169984 10.1080/14737140.2018.1518136

[11] Li D Yin N Du G. A real-world study on diagnosis and treatment of uterine sarcoma in Western China. Int J Biol Sci. 2020;16:388–95. 32015676 10.7150/ijbs.39773 PMC6990907

[12] Oliveira RC Terracciano L Cipriano MA. Primary biliary adenosarcoma of the liver-a special and new entity. Virchows Arch. 2020;477:461–6. 32219513 10.1007/s00428-020-02783-y

[13] Kaku T Silverberg SG Major FJ Miller A Fetter B Brady MF. Adenosarcoma of the uterus: a gynecologic oncology group clinicopathologic study of 31 cases. Int J Gynecol Pathol. 1992;11:75–88. 1316323

[14] Arend R Bagaria M Lewin SN. Long-term outcome and natural history of uterine adenosarcomas. Gynecol Oncol. 2010;119:305–8. 20688363 10.1016/j.ygyno.2010.07.001

[15] Krivak TC Seidman JD McBroom JW MacKoul PJ Aye LM Rose GS. Uterine adenosarcoma with sarcomatous overgrowth versus uterine carcinosarcoma: comparison of treatment and survival. Gynecol Oncol. 2001;83:89–94. 11585418 10.1006/gyno.2001.6334

[16] Duggal R Nijhawan R Aggarwal N Sikka P. Mullerian adenosarcoma (heterologous) of the cervix with sarcomatous overgrowth: a case report with review of literature. J Gynecol Oncol. 2010;21:125–8. 20613904 10.3802/jgo.2010.21.2.125 PMC2895712

[17] Tanner EJ Toussaint T Leitao MM Jr. Management of uterine adenosarcomas with and without sarcomatous overgrowth. Gynecol Oncol. 2013;129:140–4. 23283300 10.1016/j.ygyno.2012.12.036

[18] Carroll A Ramirez PT Westin SN. Uterine adenosarcoma: an analysis on management, outcomes, and risk factors for recurrence. Gynecol Oncol. 2014;135:455–61. 25449308 10.1016/j.ygyno.2014.10.022 PMC4430193

[19] Dondi G Porcu E De Palma A. Uterine preservation treatments in sarcomas: oncological problems and reproductive results: a systematic review. Cancers (Basel). 2021;13:5808. 34830960 10.3390/cancers13225808 PMC8616470

[20] Gadducci A Cosio S Romanini A Genazzani AR. The management of patients with uterine sarcoma: a debated clinical challenge. Crit Rev Oncol Hematol. 2008;65:129–42. 17706430 10.1016/j.critrevonc.2007.06.011

[21] Seagle BL Kanis M Strohl AE Shahabi S. Survival of women with Mullerian adenosarcoma: a National Cancer Data Base study. Gynecol Oncol. 2016;143:636–41. 27771166 10.1016/j.ygyno.2016.10.013

[22] Prat J. FIGO staging for uterine sarcomas. Int J Gynaecol Obstet. 2009;104:177–8. 19135669 10.1016/j.ijgo.2008.12.008

[23] Machida H Nathenson MJ Takiuchi T Adams CL Garcia-Sayre J Matsuo K. Significance of lymph node metastasis on survival of women with uterine adenosarcoma. Gynecol Oncol. 2017;144:524–30. 28109626 10.1016/j.ygyno.2017.01.012 PMC7523237

[24] Blom R Guerrieri C. Adenosarcoma of the uterus: a clinicopathologic, DNA flow cytometric, p53 and mdm-2 analysis of 11 cases. Int J Gynecol Cancer. 1999;9:37–43. 11240741 10.1046/j.1525-1438.1999.09885.x

[25] Nathenson MJ Conley AP Lin H. The importance of lymphovascular invasion in uterine adenosarcomas: analysis of clinical, prognostic, and treatment outcomes. Int J Gynecol Cancer. 2018;28:1297–310. 30044322 10.1097/IGC.0000000000001306

